# Pityriasis rosea-like drug eruption secondary to deucravacitinib

**DOI:** 10.1016/j.jdcr.2024.08.019

**Published:** 2024-09-01

**Authors:** Nisal Punchihewa, Senhong Lee, Chin-Guan Tan, Peter Foley

**Affiliations:** a Skin Health Institute, Carlton, Victoria, Australia; b The University of Melbourne, Parkville, Victoria, Australia; c Department of Dermatology, Monash Health, Victoria, Australia; d Department of Anatomical Pathology, Melbourne Pathology, Collingwood, Victoria, Australia; e Department of Dermatology, St Vincent's Hospital Melbourne, Fitzroy, Victoria, Australia

**Keywords:** cutaneous adverse reaction, deucravacitinib, drug rash, medical dermatology, pityriasis rosea

## Introduction

Deucravacitinib has emerged as an effective treatment for moderate to severe plaque psoriasis. It is accompanied by a relatively modest side effect profile compared to other oral agents.^1^ Deucravacitinib selectively binds to the regulatory domain of tyrosine kinase 2 (TYK2), an intracellular kinase that mediates signaling of interleukin-23 and other immune molecules implicated in the pathogenesis of psoriasis.^2^ It has a high specificity for TYK2 over the closely related Janus kinases, minimizing the likelihood of any unwanted “off-target” side effects.^2^

Long-term extension trials found nasopharyngitis and upper respiratory tract infections to be the most common side effects, consistent with observations in parent trials.^1^^,^^3^ Beyond acneiform eruptions and folliculitis, the distinct manifestation of any cutaneous adverse events induced by deucravacitinib remains inadequately characterized.^1^^,^^3^

Pityriasis rosea-like drug eruptions are rare and have been reported with the use of barbiturates, methopromazine, captopril, clonidine, gold, metronidazole, D-penicillamine, isotretinoin, levamisole, pyribenzamine, nonsteroidal anti-inflammatory agents, omeprazole, terbinafine, ergotamine tartrate, adalimumab, and the tyrosine kinase inhibitors imatinib and ibrutinib.^4^^,^^5^ We present a novel case of pityriasis rosea-like drug eruption secondary to treatment with deucravacitinib.

## Case report

A 51-year-old male presented with a 30-year history of chronic plaque psoriasis for consideration of systemic treatment due to increasing disease severity. Following an unremarkable preimmunosuppression screen, he was commenced on deucravacitinib 6 mg daily. Approximately 12 weeks later, after the psoriasis had resolved, the patient presented with a pruritic, papulosquamous eruption limited to the upper limbs, thighs, and trunk. The patient was systemically well and had not reported any symptoms of a preceding viral illness. There was no personal or family history of atopy.

**Fig 1:**
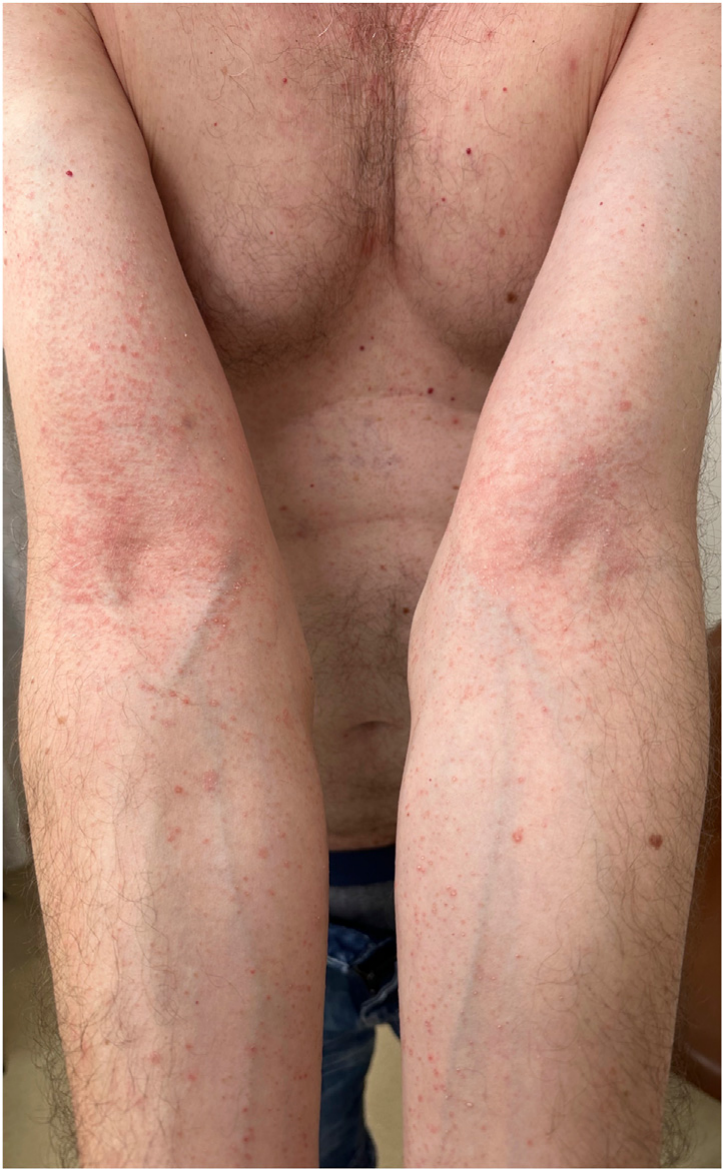
Multiple erythematous papules coalescing to form plaques with collarettes of scale distributed over the upper limbs.

**Fig 2:**
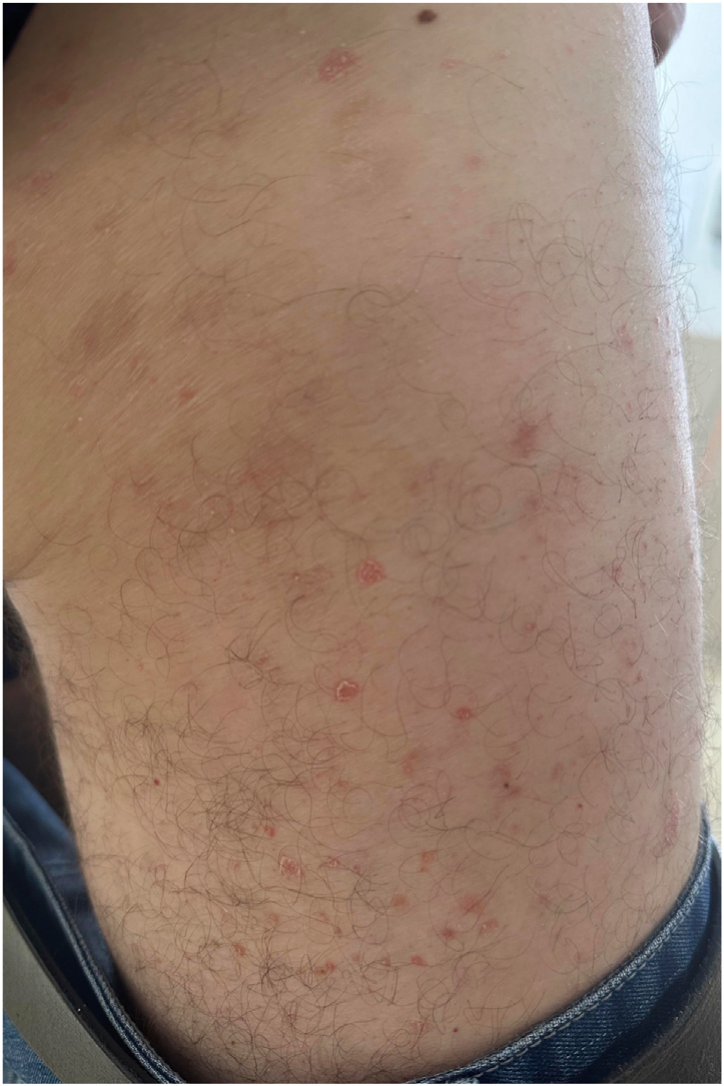
Close-up of the posterior thigh to highlight the collarette of scales.

**Fig 3:**
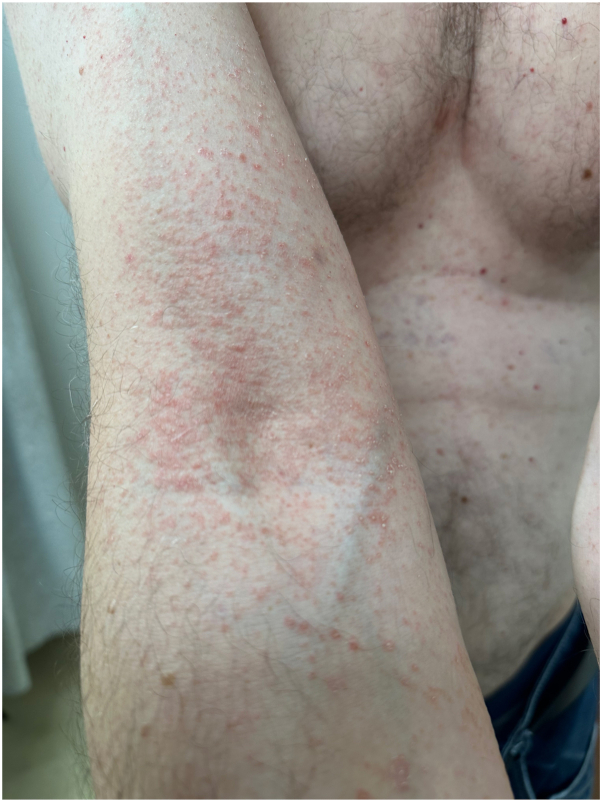
Close-up of the forearm showing multiple erythematous papules with collarettes of scale.

On examination, the patient had multiple erythematous nonfolliculocentric papules and plaques with collarettes of scale, distributed over the limbs and trunk (Fig 1, Fig 2, Fig 3). There was no herald patch or lymphadenopathy. The remainder of the general examination was normal. Punch biopsies were obtained from representative regions on the upper limbs and trunk.

**Fig 4:**
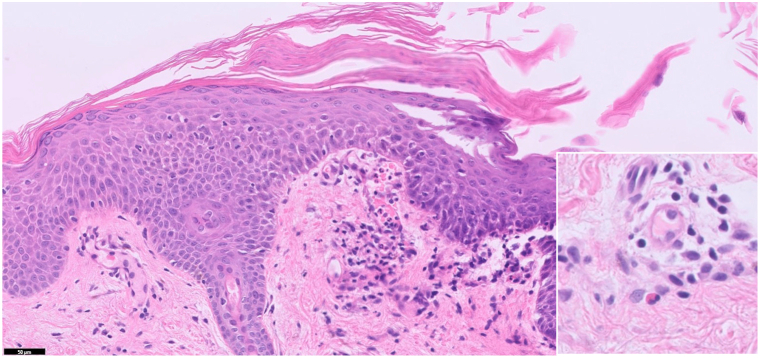
Punch biopsy from the right forearm. Spongiosis with mounds of parakeratosis, perivascular lymphohistiocytic infiltrate, and red blood cell extravasation (hematoxylin and eosin, 50×, inset highlighting scattered eosinophils).

Histopathological analysis revealed spongiosis with mounds of parakeratosis, perivascular lymphohistiocytic infiltrate, focal red blood cell extravasation, and scattered eosinophils (Fig 4). Periodic acid-Schiff staining was negative for fungal elements.

A clinico-pathological diagnosis of pityriasis rosea-like drug eruption was made. Deucravacitinib was ceased, and topical betamethasone dipropionate 0.05% ointment was prescribed. Three weeks later, there was complete resolution of the pityriasis rosea-like eruption. The patient’s plaque psoriasis was subsequently treated with apremilast 30 mg twice daily without recurrence of the pityriasiform rash.

## Discussion

Deucravacitinib, a TYK2 inhibitor, has proven to be a valuable addition to the growing array of treatments available for plaque psoriasis, demonstrating both efficacy and safety. With regard to cutaneous adverse effects, acneiform eruptions and folliculitis have been reported, but further characterization is lacking.^1^^,^^3^

To our knowledge, this is the first report of a pityriasis rosea-like drug eruption secondary to deucravacitinib. The temporal relationship observed between the initiation of deucravacitinib and the subsequent onset of the pityriasis rosea-like eruption, followed by its resolution upon discontinuation of the drug, implicates deucravacitinib as the causative agent. Additionally, the infrequent occurrence of pityriasis rosea in older individuals, coupled with the absence of a herald patch and the presence of eosinophils in the biopsy, collectively favor the diagnosis of pityriasis rosea-like drug eruption over pityriasis rosea.^6^

A potential explanation for this drug eruption involves the reactivation of latent human herpesvirus 6 and 7 infection,^4^ which may be triggered by medications that play a role in modulation of the immune system such as deucravacitinib. Human herpesvirus 6 and 7 DNA was detected in skin and tissue of patients with pityriasis rosea in contrast to rare positivity detected in control samples; however, none of these cases were drug-induced.^7^ Additionally, a pooled safety analysis of two phase III trials comparing deucravacitinib with placebo and apremilast reported that the rate of herpes zoster infection was higher in the deucravacitinib group, although this was not statistically significant.^8^ This increase was observed in patients with no prior history of herpes zoster infection or herpes zoster vaccination, suggesting that deucravacitinib may increase susceptibility to herpes viral infection or reactivation. An alternative explanation is that pityriasis rosea-like drug eruptions do not have a viral etiology and instead follow the pathogenesis of other cutaneous drug rashes, with manifestations analogous to the resemblance observed between measles and a morbilliform drug rash.^9^

The reintroduction of deucravacitinib after the pityriasis rosea-like eruption resolved was an option and would have permitted a drug “challenge”; however, our patient preferred to pursue an alternative treatment option, given the availability of other effective therapies. The precise mechanism by which deucravacitinib induced a rash in this patient remains uncertain. However, the recognition of a pityriasis rosea-like drug eruption in the context of TYK2 inhibition, coupled with previous reports associated with tyrosine kinase inhibitors and adalimumab,^4^ highlights the complex interplay between immune modulation and cutaneous manifestations.

## Conflicts of interest

Dr Foley has received grant support, and/or served on advisory boards, and/or served as a consultant, and/or has received travel grants, and/or has served as a speaker for or received honoraria from AbbVie, Akaal, Amgen, Arcutis, Argenx, Aslan, AstraZeneca, Boehringer Ingelheim, Botanix, Bristol Myers Squibb, Celgene, Celtaxsys, CSL, Cutanea, Dermira, Evelo, Galderma, GenesisCare, Genentech, GlaxoSmithKline, Hexima, Incyte, Janssen, Kymab, Leo Pharma, Lilly, Mayne Pharma, MedImmune, Melaseq/Geneseq, Merck, Novartis, Pfizer, Regeneron, Reistone, Roche, Sanofi, SunPharma, Takeda, Teva, UCB Pharma, Valeant, and ZaiLab. Drs Punchihewa, Lee, and Tan have no conflicts of interest to declare.
